# Hypertension, Obesity, and Target Organ Injury in Children: An Emerging Health Care Crisis

**DOI:** 10.1007/s11906-025-01329-4

**Published:** 2025-02-27

**Authors:** Andrew H. Tran, Aaron Walsh, Elaine M. Urbina

**Affiliations:** 1 The Heart Center, Nationwide Children’s Hospital, 700 Children’s Drive, Columbus, OH 43205 USA; 2 Department of Pediatrics, The Ohio State University, Columbus, OH USA; 3 The Heart Institute, Le Bonheur Children’s Hospital, Memphis, TN USA; 4 Department of Pediatrics, University of Tennessee Health Science Center, Memphis, TN USA; 5 The Heart Institute, Cincinnati Children’s Hospital Medical Center, Cincinnati, OH USA; 6 The University of Cincinnati, Cincinnati, OH USA

**Keywords:** Pediatric hypertension, Pediatric obesity, Target organ damage, Left ventricular hypertrophy, Pulse wave velocity, Carotid intima media thickness

## Abstract

**Purpose of Review:**

To review data regarding the association between hypertension and childhood obesity on target organ damage. We will also review data regarding the impact of intervening on hypertension and childhood obesity on target organ damage.

**Recent Findings:**

The prevalence of hypertension and obesity are rising in children despite efforts to address these risk factors. Health disparities play a role in contributing to the rise in prevalence. Hypertension and obesity promote pro-inflammatory cytokines that activate the renin-angiotensin-aldosterone system and sympathetic nervous system which result in adverse effects on blood pressure regulation and renal function. Adverse cardiac, vascular, renal, neurocognitive, and retinal changes can be seen with elevated blood pressure. Recent intervention studies are few, but adequate treatment of hypertension and obesity can result in improvement in target organ damage.

**Summary:**

Hypertension and obesity have significant impacts upon target organs. Interventions to decrease blood pressure and treat obesity are associated with reductions in left ventricular hypertrophy, improvement in measures of systolic and diastolic function, and improvement in renal outcomes. Appropriate screening and management of these conditions can lessen potential future cardiovascular impact.

## Introduction

Both hypertension and obesity are known independent risk factors for cardiovascular (CV) events in adulthood. A recent study estimates that cutting back on sodium intake and adequately controlling hypertension could delay approximately 35 million deaths in women and 45 million deaths in men over 25 years [1]. Similarly, treating obesity results in significant improvements in CV risk profile in adults [2, 3]. When examining outcomes in children, measures like CV events or mortality are less useful when evaluating the effect of hypertension and obesity. However, markers of target organ damage (TOD) can serve as more proximal outcome measures showing that the effects of hypertension and obesity are not just limited to adulthood but also result in adverse impacts during childhood. This review’s primary focus will be on the impact of hypertension on children while including the interplay of obesity. We will focus on recent literature detailing the current epidemiology of hypertension and obesity. Additionally, we will examine the mechanisms for TOD and the association between hypertension, obesity, and TOD. Lastly, we will describe literature regarding the impact of interventions on TOD and future research needs.

## Epidemiology of Hypertension and Obesity

High blood pressure (BP) is a modifiable risk factor for the development of atherosclerotic CV disease. Rates of elevated BP and hypertension among youths have been tracked over several decades and have paralleled the increasing prevalence of childhood obesity. New diagnostic criteria were proposed in 2017 to establish age, sex, and height-specific percentile tables for children and to align adolescent (≥ 13 years) BP categories with those of adults. This enabled a streamlined approach to the identification of hypertension and to make cutoffs congruent with those in adults which have shown associations with CV outcomes. Despite a period of decreasing rates of hypertension in the early 21st century, rates of pediatric hypertension are on the rise with the prevalence of abnormal BP in adolescents to be roughly 15%, with 2–5% of those being classified as hypertensive [4].

Childhood obesity remains a public health crisis and a key driver of cardiometabolic disease leading to atherosclerotic CV disease. Rates of obesity have risen over the last decade, increasing from 16.9% in 2011 to almost one in five youths by 2020 [5]. Risk factors for obesity include age, non-White race, and lower family income [6]. Significant increases in prevalence of obesity have been seen in children aged 2–5 years, Mexican Americans, non-Hispanic Blacks, and the rate of severe obesity (BMI-for-age ≥ 120% of the 95th percentile).

Health disparities amongst differing ethno-racial groups and household income are key drivers to the promotion of poor cardiometabolic profiles. The complex interplay between environmental, economic, political, and sociocultural influences on rates of obesity have recently been described [7]. Limited access to quality foods, lack of convenient and safe areas for exercise, and the wealth gap disproportionately affect minority groups. Indeed, obesity prevalence is highest amongst Hispanic (26.2%) and non-Hispanic black (24.8%) children and adolescents [6]. Rates of high BP are also higher among Hispanic and African Americans [8] as well as those from lower income families [9].

## Mechanisms of TOD from Hypertension and Obesity

**Fig. 1:**
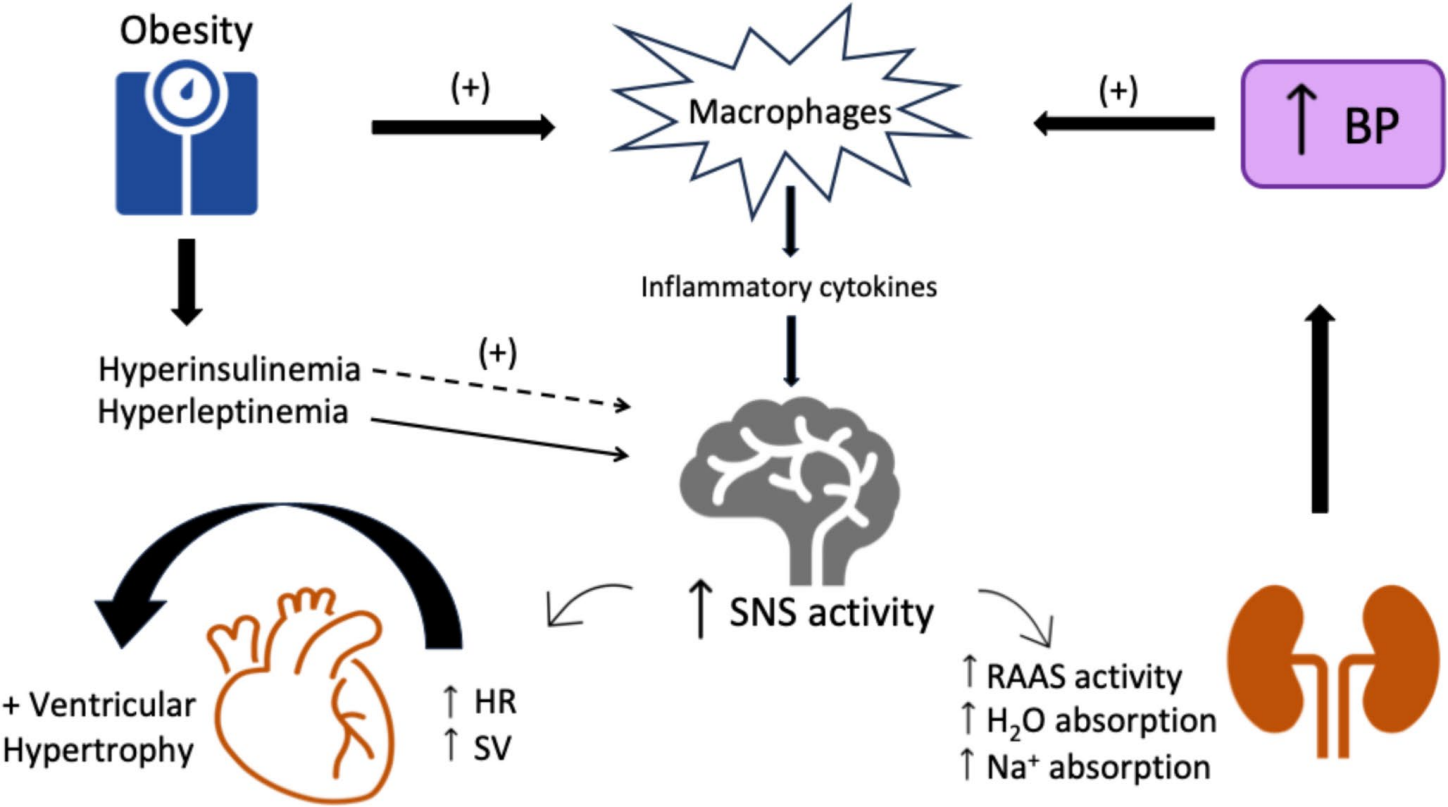
Mechanisms of target organ damage from hypertension and obesity. Dotted arrow denotes possible effect BP = blood pressure; HR = heart rate; PVR = pulmonary vascular resistance; SV = stroke volume; RAAS = renin-angiotensin-aldosterone system; H_2_O = water; Na^+^ = sodium

Hypertension and obesity are well established risk factors for CV disease. Both conditions contribute to pathologic adaptations in cardiomyocyte architecture which can jeopardize the balance of oxygen supply and consumption leading to risk of myocardial infarction and heart failure. Increased afterload exerted by high BP forces myocytes to hypertrophy in order to reduce wall tension. This pressure-loaded state results in concentric hypertrophy of the myocardium as a compensatory mechanism that is effective in maintaining proper hemodynamics but eventually leads to cardiac dysfunction due to development of abnormal ventricular wall remodeling, fibrosis, and altered coronary microcirculation [10]. Obesity also alters cardiomyocytes by exerting a volume load characterized by increased stroke volume and cardiac output that can lead to eccentric and concentric left ventricular hypertrophy (LVH) [11–13]. These cellular changes are mediated by complex interactions between the autonomic nervous system, circulating hormones, inflammation, and the renal system ultimately leading to alterations in BP control and deleterious cardiac remodeling (Fig. 1) [10, 11]. The concept of cardiac power output ([Cardiac Output * Mean Arterial Pressure] / 451 = Cardiac Power Output) is typically used as an outcome measure for patients with heart failure [14, 15] but can serve as a useful way to visualize the interplay between arterial pressure and cardiac output.

Both hypertension and obesity are chronic inflammatory states. The cardiac remodeling found in these conditions can be attributed to the activation of circulating macrophages which release pro-inflammatory cytokines such as TNF-alpha and IL-6 which promote signaling through the MAP kinase and Nf-kB pathways [16]. In obesity, this process is partially mediated by progressive hypoxia inside enlarging adipocytes. In hypertension, oxygen supply and demand imbalances secondary to increased cardiac workload triggers the activation of transcription factors that promote the pro-inflammatory macrophage phenotype [17]. Together, obesity and hypertension-induced activation of macrophages promotes release of inflammatory cytokines with downstream activation of the sympathetic nervous system and renin-angiotensin-aldosterone system (RAAS) with deleterious effects on BP regulation and renal function. A disproportionate increase in aldosterone to renin can also impact BP. Individuals with high aldosterone to renin ratio were found to have significantly higher BP compared to those with low aldosterone to renin ratios [18]. Furthermore, aldosterone is associated with the development of insulin resistance, inflammation, endothelial dysfunction, and arterial stiffness which could further contribute to the development of hypertension [19].

Leptin and adiponectin are cytokines released by adipocytes that play a role in appetite regulation and insulin sensitivity, respectively. Leptin exerts its anorexigenic effects through autonomic regulation and stimulation of the sympathetic nervous system. Obese individuals have been shown to develop a resistance to leptin resulting in a chronically overstimulated sympathetic nervous system and subsequent hypertension [20]. Levels of the anti-inflammatory protein adiponectin are reduced in obesity [21]. Overeating also stimulates the sympathetic nervous system via direct effects of glucose and insulin on the central nervous system. Together with the central effects of insulin and leptin, persistent sympathetic activation results in chronic alpha-receptor mediated increase in peripheral vascular resistance that preferentially affects the renal system [22].

## Association of Hypertension, Obesity, and TOD

The adverse physiologic changes associated with hypertension and obesity lead to subclinical markers of end-organ damage. The following sections detail the impact of hypertension and obesity in various organ systems (Table 1).

**Table 1:** Associations between hypertension and target organ damage

Organ System	Outcomes
Cardiac	↑ LVH ↑ LVMI Altered left ventricular strain Impaired diastolic function
Vascular	↑ Carotid-femoral pulse wave velocity ↓ Aortic distensibility ↓ Aortic compliance ↑ Carotid intima-media thickness
Renal	↑ Albuminuria ↓ Glomerular filtration rate
Brain	Worse neurocognitive measures Adverse impact on memory Worse learning measures
Eye	Adverse retinal vascular changes

LVH = left ventricular hypertrophy; LVMI = left ventricular mass index

### Cardiac & Vascular

LVH and increased arterial stiffness are measurable consequences of hypertension and obesity that are present even during the childhood years. A recent meta-analysis found that children with ambulatory hypertension had higher risk for LVH and increased left ventricular mass index (LVMI) compared to normotensive children [23]. Furthermore, even at BP levels below current thresholds for hypertension, children show evidence of subclinical systolic and diastolic dysfunction [24]. Findings from a recent meta-analysis also support alterations in left ventricular strain and impaired diastolic function in children with primary hypertension compared to controls [25]. Children with obesity have significantly higher prevalence of LVH and impaired diastolic function compared to children without obesity [26]. Children with both overweight/obesity and elevated BP also demonstrated left ventricular diastolic dysfunction [27].

Aortic pulse wave velocity (PWV) is a common measurement of pulse transit time and is a marker of central vascular stiffness. PWV values increase with vessel stiffness. Increased PWV has been shown to be a predictor of CV morbidity and mortality in adults [28]. Haley et al. evaluated 382 youth who were grouped into low- (systolic BP < 75th percentile), mid- (≥ 80th and < 90th percentile), and high-risk BP categories (≥ 90th percentile) with carotid-femoral pulse wave velocity (cfPWV), aortic distensibility, distensibility coefficient, and aortic compliance [29]. Carotid-femoral PWV significantly increased across the BP groups, and the low-risk BP group had higher (healthier) values of aortic distensibility and compliance compared to the mid- and high-risk BP groups. BP was a significant independent determinant of arterial parameters along with age, sex, adiposity, and low density lipoprotein cholesterol. These findings of arterial changes are important as they were also independently associated with measures of cardiac function and albuminuria. Supporting these findings of early vascular changes, Chung et al. found in a meta-analysis that children with ambulatory hypertension had significantly higher risk of elevated PWV and cIMT compared to normotensive controls [23].

There is also a relationship between vascular health and cardiac changes. Urbina et al. demonstrated that youths with high BP showed evidence of increased PWV and that arterial stiffness predicted cardiac diastolic dysfunction and renal microvascular dysfunction [29]. Cardiac systolic performance may also be impaired in youths with increased arterial stiffness [29, 30]. Similar results have been described in children with obesity. In a meta-analysis of over fifteen studies on arteriosclerosis in children and adolescents with obesity, all but two studies showed greater increased arterial stiffness in those with obesity [31].

### Renal

Hypertension can adversely affect renal function. The presence of hypertension is associated with kidney injury even in the absence of pre-existing renal disease [32, 33]. While data are limited in children regarding the association between hypertension and microalbuminuria, more robust data is available in adults [32] and long-term cohort study data show associations between childhood hypertension and adult albuminuria [34]. A recent pediatric study examined children with cardiovascular risk factors (CVRFs) including hypertension, obesity, dyslipidemia, and insulin resistance and evaluated the association between the number of CVRFs and measures of cardiac, vascular, and renal TOD [35]. Children with > 2 CVRFs had significantly different albumin to creatinine ratio versus children with 0 CVRFs. Notably, BP was significantly associated with all measures of TOD.

### Neurocognition

The accumulation of CV risk factors also has neurocognitive effects. The Young Finns Study assessed a cohort of children into adulthood and found that cumulative exposure to high systolic BP, elevated total cholesterol, and smoking during childhood was associated with worse neurocognitive measures during midlife [36]. Memory and learning measures were particularly affected. However, the adverse impact of hypertension on neurocognition can also be seen during early adulthood [37] and even childhood [38, 39].

### Retinal

Retinal vasculature can provide an early look into the effects of hypertension on children. Children with systolic hypertension have narrower central retinal arteriolar equivalents (CRAE) versus children with normal or elevated BP [40]. The authors also evaluated the effect of BMI on retinal measures and found that children with both hypertension and overweight/obesity had the most adverse retinal measures. Wider central retinal venular equivalents (CRVE) and decreased arteriolar fractal dimensions were also associated with higher BP. Another study evaluated CRAE and CRVE longitudinally over 4 years in children and found that those with high systolic or diastolic BP at baseline had narrower CRAE at follow-up [41]. The converse was also true with narrower CRAE at baseline predicting later development of higher systolic BP.

### Lifecourse

The impact of pediatric hypertension extends into adulthood. A large cohort study of school children was followed over 30 years in China and found that isolated diastolic hypertension during childhood was associated with increased arterial stiffness and albuminuria during adulthood [34]. Additionally, the long-term burden of elevated diastolic BP was assessed using area under the curve (AUC) techniques and was found to be associated with arterial stiffness, albuminuria, and LVH. BP trajectories are another method of evaluating BP over the lifecourse. An extension of the prior cohort study found that individuals that had persistently high BP or who had an increasing BP trajectory from childhood into adulthood had higher risk for LVH and increased carotid intima-media thickness (cIMT) [42]. Overall, these findings show the significant impact of pediatric hypertension over the lifecourse.

## Effects of Intervention on TOD

Adequate treatment of hypertension can reduce measures of TOD such as LVH in adults [43, 44]. Similar findings have been shown in children with adequate hypertension treatment [32, 45, 46]. However, compared to the adult literature, there are relatively fewer pediatric studies examining the effects of interventions on TOD, particularly when compared to the number of pediatric studies examining the presence of TOD in hypertension. As such, this review will discuss some key past studies to provide context and then move into discussion of more recent studies. Kupferman et al. found that children with hypertension and LVH that were treated with angiotensin-converting-enzyme (ACE) inhibitors had significant regression in LVMI on follow-up echocardiograms [47]. Improving obesity can also result in improvements in BP. Holm et al. evaluated children with obesity undergoing a 12-week weight loss program and found that by the end of the intervention, there were significant improvements in both BMI and BP [48]. Over long-term follow-up, weight regain was accompanied by BP increases, particularly for diastolic BP. For even higher risk youth with severe obesity and type 2 diabetes mellitus, medical treatment alone with lifestyle and medication was ineffective in reducing the prevalence of elevated BP, but bariatric surgery greatly reduced prevalence rates [49]. Improvements in LVMI, diastolic function, and cardiac geometry are also seen in adolescents with severe obesity after bariatric surgery alongside the BP improvements [50]. BMI reduction in obese children is also associated with improvements in global longitudinal strain, a measure of systolic function [51].

Diet and lifestyle therapies are typically the first-line treatment for children with elevated BP (Table 2). Genovesi et al. examined the effectiveness of diet and lifestyle therapies on children with excess weight, elevated BP, or both conditions [52]. They found that there was a significant decrease in the prevalence of hypertension, obesity, and LVH at follow-up compared to baseline measurements. LVMI as a continuous measure was also significantly decreased at follow-up. While antihypertensive medication is an important part of managing hypertension, this study shows that targeted diet and lifestyle therapy can be an effective approach in managing hypertension and obesity in children.

**Table 2:** Effects of intervention on target organ damage

Intervention	Effects
Diet and lifestyle	↓ BP ↓ BMI ↓ LVH
Decreasing BP	↓ LVMI Can still have altered diastolic function versus normotensive patients Slower progression of kidney disease Possible neurocognitive benefits
Decreasing BMI	↓ BP ↓ LVMI Improved diastolic function Improved cardiac geometry

BMI = body mass index; BP = blood pressure; LVH = left ventricular hypertrophy; LVMI = left ventricular mass index

Another study by Kaplinski et al. evaluated the effect of antihypertensive medication on TOD in children with hypertension [53]. While hypertension, obesity, and race were found to be associated with LVMI on univariate analysis, obesity was the only significant predictor of LVMI on multivariate analysis. These results support findings of other studies suggesting that obesity has a significant impact on LVMI compared to other factors including hypertension [32, 54]. Of note, Kaplinski et al. also found that even when patients with hypertension were adequately treated, they still had worse subclinical LVMI and diastolic function findings compared to normotensive youth. These findings are important because it shows that even with treatment there can be subclinical differences in cardiac structure and function that may increase future CV risk. Strategies incorporating TOD assessment as part of antihypertensive treatment titration rather than just using BP goals may help to prevent worsening TOD. Alternatively, more aggressive BP goals may be needed to prevent TOD development.

The impact of antihypertensive treatment on TOD in children with chronic kidney disease is significant. The landmark study by the ESCAPE Trial Group found that intensified BP control with mean arterial pressure (MAP) < 50th percentile resulted in decreased worsening of glomerular filtration rate and progression to end-stage renal disease compared to conventional BP control (MAP of 50-95th percentile) [55]. More recently, Byfield et al. performed a subset analysis of participants from the Chronic Kidney Disease in Children (CKiD) study to evaluate the effect of antihypertensive treatment but focused on medication nonadherence in association with markers of TOD and ambulatory blood pressure monitor (ABPM) parameters [56]. Nonadherence was defined as missing any doses of antihypertensive medication in the 7 days prior to the study visit. They found that baseline nonadherence was not associated with cardiac or renal TOD measures at follow-up. Furthermore, baseline nonadherence was not associated with ABPM parameters. These findings are unexpected given data such as from the ESCAPE Trial suggesting adequate BP control improves TOD measures [55]. However, it is possible that the measure of baseline nonadherence was not specific enough in this patient population to assess true long-term nonadherence.

There have been very few recent studies published examining the effect of hypertension intervention on neurocognitive outcomes. The most recent was published in 2018 by Lande et al. who performed neurocognitive testing in hypertensive versus normotensive youth at baseline and at 1 year follow-up [57]. The testing encompassed measures of general intelligence, attention, memory, executive function, and processing speed. Patients with hypertension were treated with antihypertensive medication, and there was a significant improvement in BP in the hypertensive group over time. When examining neurocognitive testing results, there was improvement in both groups over time but no significant difference between groups leading the authors to conclude that the improvements in testing were likely related to age and improved familiarity with the tests. However, when looking specifically at patients who had persistent ambulatory hypertension at follow-up, there was lack of improvement in subtests of the Rey Auditory Verbal Learning Test and only limited improvement in the Grooved Pegboard test suggesting possible effects of sustained hypertension on neurocognition. It is also possible that with a longer follow-up time frame, more significant differences in neurocognition may emerge.

## Research Needs and Future Directions

There are clear areas of research need for children with hypertension. While there have been numerous studies evaluating the presence of TOD in children with hypertension, there are comparatively fewer studies assessing the impact of intervention on TOD. Additionally, more longitudinal studies of the impact of intervention on TOD in children are needed including both pharmacologic and non-pharmacologic management of hypertension. The impact of social determinants of health on hypertension in children should also be examined as these factors have been shown to be associated with increased CV risk in adults and other risk conditions in children. Furthermore, as there is robust and increasing evidence of the impact of hypertension in children, more implementation science studies are needed to determine the best methods of delivering effective care for these children.

## Summary

Children with hypertension and obesity have increased risk for TOD in multiple organ systems. These same measures of TOD are associated with CV events in adults highlighting the importance of appropriate screening and management of hypertension and obesity in children. Future studies are needed to further assess the impact of adequate treatment on TOD, the influence of social determinants of health on these risk factors, and implementation science in more effectively bringing evidence-based care to children.

### Key References

**4. Falkner B, Gidding SS, Baker-Smith CM, Brady TM, Flynn JT, Malle LM, et al. Pediatric primary hypertension: An underrecognized condition: A scientific statement from the American heart association. Hypertension [Internet]. 2023;80:e101–11. Available from: https://pubmed.ncbi.nlm.nih.gov/36994715/.

**This scientific statement summarizes the current knowledge of pediatric primary hypertension including diagnostic and management strategies as well as long term sequelae highlighting the influence that increasing adiposity has had on the prevalence of childhood hypertension.**

*29. Haley JE, Woodly SA, Daniels SR, Falkner B, Ferguson MA, Flynn JT, et al. Association of blood pressure-related increase in vascular stiffness on other measures of target organ damage in youth. Hypertension [Internet]. 2022 [cited 2024 Sep 5];79:2042–50. Available from: https://pubmed.ncbi.nlm.nih.gov/35762327/.

**This study highlights the association between blood pressure and arterial stiffness and the association between arterial stiffness, cardiac diastolic dysfunction, and renal microvascular dysfunction.**

**35. Price JJ, Urbina EM, Carlin K, Becker R, Daniels SR, Falkner BE, et al. Cardiovascular risk factors and target organ damage in adolescents: The SHIP AHOY study. Pediatrics [Internet]. 2022 [cited 2024 Sep 5];149. Available from: https://pubmed.ncbi.nlm.nih.gov/35502610/.

**This study demonstrates the association between the number of cardiovascular risk factors and target organ damage in children.**

**42. Zheng W, Mu J, Yan Y, Chu C, Su X, Man Z, et al. Associations of blood pressure trajectories in early life with target organ damage in midlife: a 30-year cohort study. Hypertens Res [Internet]. 2023 [cited 2024 Sep 5];46:2613–21. Available from: https://pubmed.ncbi.nlm.nih.gov/37553520/.

**This study showed that children with persistently high blood pressure or who had increasing blood pressure trajectory into adulthood had increased risk for adult target organ damage.**

*52. Genovesi S, Tassistro E, Giussani M, Antolini L, Lieti G, Orlando A, et al. Association between lifestyle modifications and improvement of early cardiac damage in children and adolescents with excess weight and/or high blood pressure. Pediatr Nephrol [Internet]. 2023 [cited 2024 Oct 15];38:4069–82. Available from: https://pubmed.ncbi.nlm.nih.gov/37349569/.

**This study found that targeted diet and lifestyle changes can result in improvement in prevalence of hypertension, obesity, and left ventricular hypertrophy in children.**

## Data Availability

No datasets were generated or analysed during the current study.
